# FES-Induced Cycling in Complete SCI: A Simpler Control Method Based on Inertial Sensors

**DOI:** 10.3390/s19194268

**Published:** 2019-10-01

**Authors:** Benoît Sijobert, Ronan Le Guillou, Charles Fattal, Christine Azevedo Coste

**Affiliations:** 1 Institut National de Recherche en Informatique et en Automatique (INRIA), Université de Montpellier, 34095 Montpellier, France; benoit.sijobert@gmail.com (B.S.); ronan.le-guillou@inria.fr (R.L.G.); 2 CRRF La Châtaigneraie, 95180 Menucourt, France; cfattal@lachataigneraie.fr

**Keywords:** FES cycling, inertial sensors, neurorehabilitation

## Abstract

This article introduces a novel approach for a functional electrical stimulation (FES) controller intended for FES-induced cycling based on inertial measurement units (IMUs). This study aims at simplifying the design of electrical stimulation timing patterns while providing a method that can be adapted to different users and devices. In most of studies and commercial devices, the crank angle is used as an input to trigger stimulation onset. We propose instead to use thigh inclination as the reference information to build stimulation timing patterns. The tilting angles of both thighs are estimated from one inertial sensor located above each knee. An IF–THEN rule algorithm detects, online and automatically, the thigh peak angles in order to start and stop the stimulation of quadriceps muscles, depending on these events. One participant with complete paraplegia was included and was able to propel a recumbent trike using the proposed approach after a very short setting time. This new modality opens the way for a simpler and user-friendly method to automatically design FES-induced cycling stimulation patterns, adapted to clinical use, for multiple bike geometries and user morphologies.

## 1. Introduction

Functional electrical stimulation (FES) refers to the activation of muscle contractions by the application of short electrical pulses. In some conditions, the FES technique can be used in paralyzed muscles to provide functional or therapeutic benefits. In individuals with spinal cord injury (SCI), a clinical use of FES to activate lower limb muscles has proven to reduce risk factors for cardiovascular disease and decrease other risks related to a prolonged sitting posture (e.g., pressure sores, atrophy) by maintaining muscular activity [1,2]. FES-assisted cycling and rowing have been demonstrated to be particularly efficient in preventing psychological and physical function deterioration by stimulating thigh and glutei muscles to propel a cycling or rowing machine [3]. While increasing self-esteem and wellness [1], FES-induced cycling and rowing exercises also positively affect the cardiopulmonary system [4,5], bone mineral density [6] and muscle strength [7,8]. Literature on FES cycling is particularly abundant and reflects a growing interest over the past thirty years [9].

In most of these studies, FES cycling is achieved while the user is installed in a fixed ergocycle at a rehabilitation center. However, multiple studies have demonstrated that adding a recreational dimension to this physical exercise could improve the attractiveness of FES-assisted training and increase the psychological wellness of the user [2]. This can be achieved by using an instrumented mobile tricycle, designed for outdoor use, and some of these devices are commercially available. In most of them, the stimulation relies on a preset pattern, alternatively sending stimulation to quadriceps, hamstrings, and glutei muscles, depending on the crank angle, estimated by an encoder. However, adaptation to different users requires manual tuning of the stimulation patterns. An individual tuning has to be performed for each user, depending on his/her seating position and anthropometry, the number of muscles being stimulated, and the bike geometry. Various studies have investigated the possibility of using different sensors inputs (Electromyography [10], inertial sensors [11,12], oxygen measurement [13], pedal forces [14]) to automatically design and optimize cycling stimulation patterns and to enhance device usability. Other studies have investigated novel control strategies (neural network [15], fuzzy logic [16]) to improve cycling performances: Maximum covered distance or force and complex FES controllers have been proposed but have not provided significant improvement of the functional results compared to more simple ones [17]. Other works have tried to improve the performance by changing the stimulation frequency [18], the recruited muscles [19], cadence or the mechanical design [20,21].

Using inertial measurement units (IMUs) to analyze gait and posture, as well as to control electrical stimulation delivery, has been deeply investigated in prior studies led by the authors [22,23,24,25,26]. While inertial sensors have been previously considered in a large number of applications [27,28] they have only recently been applied in FES-cycling [12,29].

In the work by Wiesener et al. [11], the authors used a two-dimensional geometric model for the lower limbs, the knee and hip absolute angles were estimated from four IMUs located on the thighs and shanks, and then transformed into a normalized range [0;1]. Instead of using the crank-angle-based stimulation pattern, a cycling percentage (CP) was used to define the stimulation pattern for each leg. CP defines two ranges that are easily identifiable from the knee angles: Flexion and extension. Depending on these two phases, different muscles contributions were activated to produce a positive torque. However, the method proposed in that work relied on a geometric model of the lower limbs and a crank sensor to estimate the real pedal position. Several solutions have been investigated by Wiesener et al. to counteract the effects of sliding in seat position and IMU placement on method robustness [29]. This approach was successfully used during the Cybathlon competition in 2016 [11]. Due to the cycling and FES devices’ independence, the same setting and stimulation pattern can be used for different cycling equipment, used by the same pilot.

Most of the existing solutions are globally complex and time consuming. They highlight the inherent difficulties in designing a stimulation pattern able to produce active cycling. On the other hand, it is essential to keep in mind that most of these applications are intended for clinical or personal use. The final users are either medical practitioners or the pilots themselves, and providing intuitive interfaces to adapt the stimulation patterns in a simple and quick manner would probably be valuable in FES-cycling practice. Based on the arguments of Wiesener et al., and on our personal experience in FES cycling [30,31,32], we present here a pilot study focused on the further simplification of the control of stimulation delivery while ensuring a maximum ease of use, adaptability and tailored solution for the user using two inertial sensors only and no a priori information on cycling device geometry, nor pilot anthropometric data.

## 2. Materials and Methods

### 2.1. Subject

One participant (male; 48 y), the so-called pilot, with complete spinal cord lesion (20-years old spinal cord lesion T3 ASIA motor score 50) was included in this protocol. The study was approved by the French national ethics committee (Comité de Protection des Personnes) and the participant gave his written informed consent. Prior to joining the study, the pilot got acquainted with the stimulation by taking part in a 2-year training FES-cycling protocol [32].

### 2.2. Material

**Figure 1:**
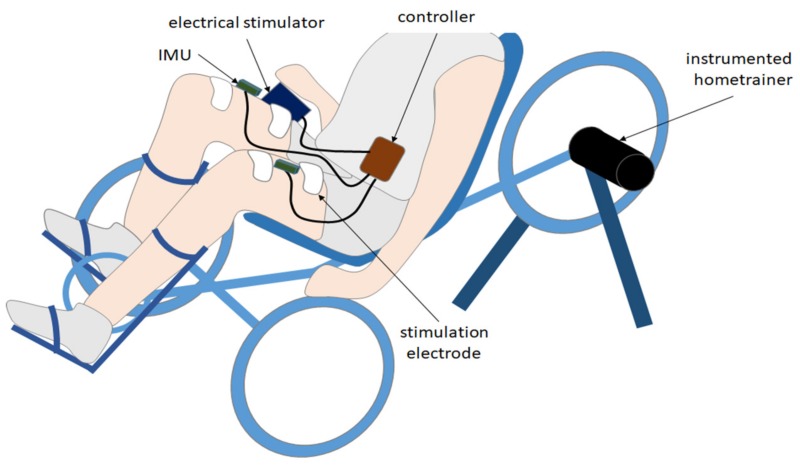
Experimental setup designed to validate the proposed approach. The pilot was equipped with 2 IMUs located on each thigh. IMUs were wired to a Raspberry Pi3© (the controller) communicating with an electrical stimulator. An instrumented home trainer was set on the rear wheel to monitor power output during the experiment.

Installed on a tricycle (ICE Adventure ©, Falmouth, UK) with a fixed-gear rear wheel, the pilot was equipped with two inertial measurement units (IMU, Bosch© BNO055, Gerlingen, Germany) located on each thigh (Figure 1). IMUs were wired to a Raspberry Pi3© (i.e., the controller). Each IMU was embedded with a high-speed ARM Cortex-M0 based processor and a Kalman filter directly providing the quaternion estimation needed to compute angles at a 100 Hz sampling rate. The controller was also connected to an electrical stimulator (BerkelBike©, Sint-Michielsgestel, Netherlands) located on the bike. The controller sent the start/stop command messages to the stimulator. Two stimulation channels were active and sent current pulses to both quadriceps by means of two pairs of skin electrodes. An instrumented home trainer, specifically designed to record weak power (<200 W) while ensuring a minimum accuracy of 0.5% (rotating torque meter Scaime© TSR 2300, Juvigny, France), was set on the rear wheel to monitor power output during the experiment.

### 2.3. Algorithm

IMU data were processed online to detect the ‘Peak Thigh Flexion’ (PTF) and ‘Peak Thigh Extension’ (PTE) chosen as the start and stop events of the quadriceps stimulation. Thigh tilting angles (θ) relative to the horizontal were computed for each new sample (n) using the method detailed in [33], by combining the embedded sensor fusion algorithm with trigonometric transformations to obtain the thigh tilting angle as a Euler angle.

**Figure 2:**
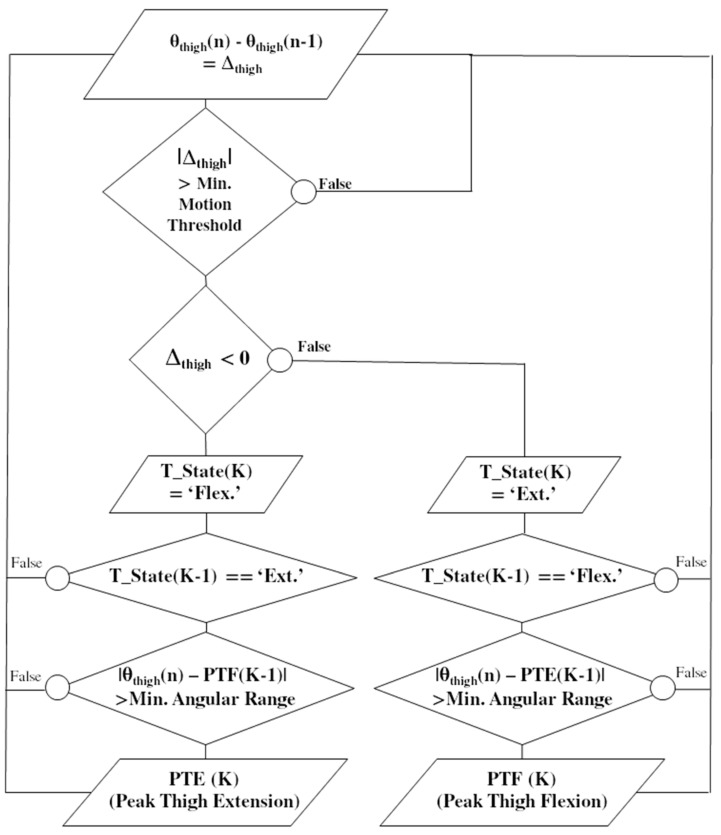
Flowchart illustrating the real-time detection algorithm of the ‘Peak Thigh Flexion’ (PTF) and ‘Peak Thigh Extension’ (PTE) events, corresponding to the maximum and minimum tilting of the thigh during one crank cycle. The thigh tilting angles [θ] are computed for each new sample [n]. The actual state of the thigh (T_state (K)) is updated depending on a flexion or extension is detected, in relation to the last known state (K−1). By combining gradients and IF–THEN rules with preset thresholds, events are robustly detected.

As illustrated in Figure 2, a simple IF–THEN rules algorithm combining gradient detection with preset thresholds was designed to reliably detect PTF and PTE. For each new sample, a test is first performed to check if the difference between two consecutive computed angles is higher than a minimum threshold (set to 2°), which aimed to get rid of false positive in the case of small motions, such as tremors. A second test is then performed to monitor the gradient sign and update a state variable, T_State; if negative, the motion is considered as a flexion and if positive the motion is considered as an extension. Two tests are then computed: (1) if the state variable changes from flexion to extension and (2) if the actual thigh tilting angle is above a minimum range of motion (set to 20°) relative to the last PTE, then a PTF event is detected. A full passive cycle rotation was needed to identify two initial PKT and PKE events for each leg before starting the detection and automatically triggering the stimulation. For each quadriceps, stimulation started at PTF until the detection of PTE. A parameter enables to adjust the stimulation onset a few degrees before or after PTF.

### 2.4. Protocol

The stimulation electrodes were positioned over quadriceps (vastus medialis and rectus femori) and the two inertial sensors were fixed on the thigh with rubber bands. After this, the participant was transferred from his wheelchair to the bike, with minimum assistance in order to guarantee his safety regarding sharp metallic elements. Feet were stabilized using calf supports.

**Figure 3:**
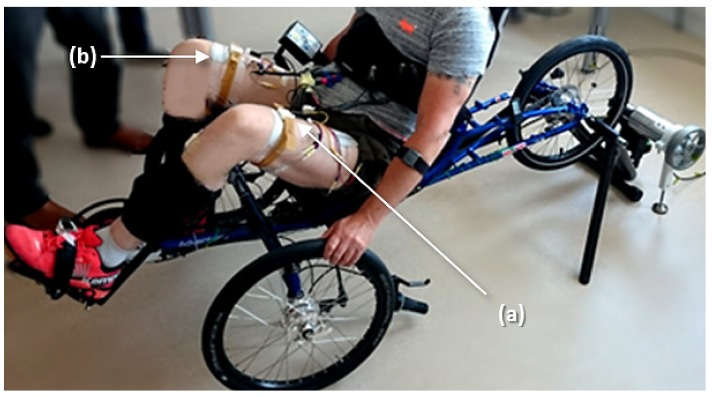
Home trainer cycling. The participant was installed on a recumbent trike and was equipped with 2 IMUs (**a**) connected to the controller and 2 pairs of surface electrodes (**b**) connected to the electrical stimulator. An instrumented home trainer enabled to measure the power produced.

During the first part of the experiment, the tricycle was installed on the home trainer support (Figure 3) adjusted with a negligible rolling resistance. After a first passive cycle where the experimenter moved the pedals manually, the onset of the stimulation in the different channels was automatically triggered by the previously-described algorithm. The participant could adjust the stimulation intensity by pressing buttons directly on the stimulator until the cycling movements was totally induced by muscle contraction and the experimenter could stop accompanying the movement. A warm-up stimulation session using a crank-angle-based stimulation pattern at 20 Hz was performed before testing the algorithm. For the rest of the protocol, the frequency was set to 30 Hz. The electrical stimulator was pre-programmed to deliver charge-balanced biphasic pulses, with a pulse width of 400 µS per phase. The stimulation intensity was limited to a maximum of 150 mA and gradually increased from 20 mA by the participant in order to obtain an active cycling movement. Based on the state-of-the-art, the most efficient cycling cadence in these experimental conditions has been proven to be around 47 rpm, which corresponds to a velocity of 5.6 km/h. The participant could adjust the stimulation intensity by pressing ‘plus’ and ‘minus’ buttons on the stimulator box in order to maintain this speed.

For the second part of the experiment, the bike was removed from the home trainer and installed in a 40-meter corridor.

## 3. Results

**Figure 4:**
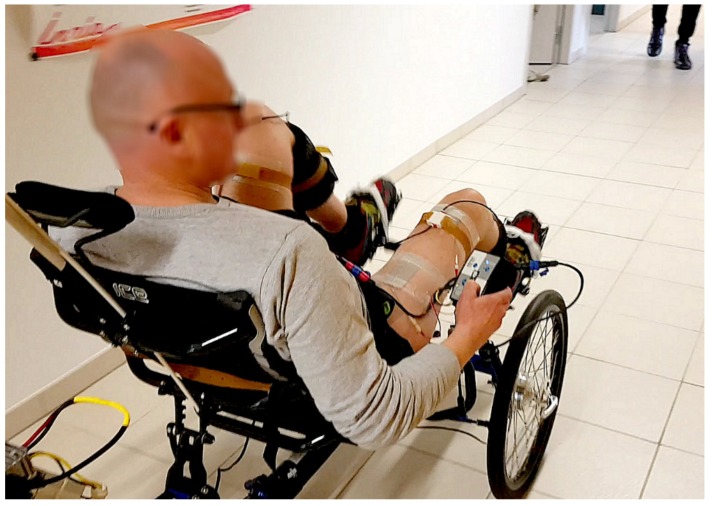
Overground cycling. The participant was able to propel the recumbent trike over a 40-meter corridor.

The algorithm presented previously was tested successfully. The participant was able to cycle on the recumbent trike in stationary conditions (i.e., installed on the home trainer, Figure 3), as well as to propel the trike in mobile conditions (i.e., rolling on a flat surface in a corridor, Figure 4).

**Figure 5:**
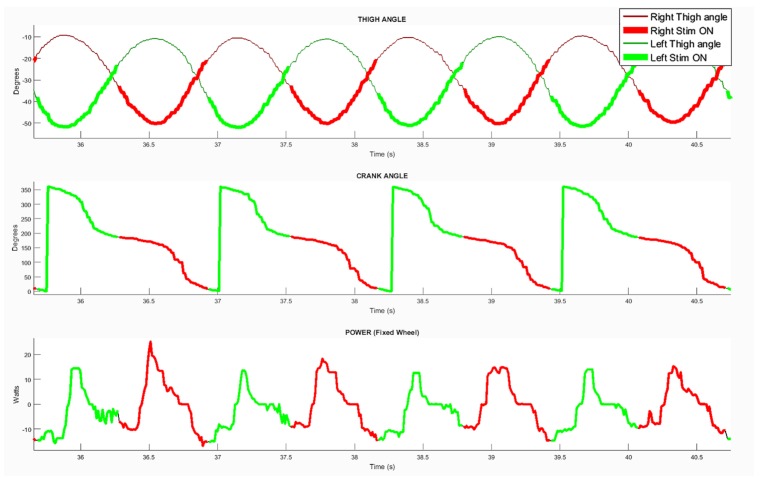
Data sample illustrating the results over four pedaling cycles in home trainer conditions. (**Top**) Left (green) and right (red) thigh tilting angles; (**middle**) crank angle; (**bottom**) developed power. The two stimulation channels activation are highlighted.

The parameter enabling an offset before stimulation onset had to be adjusted to optimize the pedaling. After testing multiple values, the best efficiency was obtained with a value of 15° before PTF. In order to quantify the detection accuracy and compare it to the crank angle (i.e., the usual stimulator input), several data-points were monitored over crank cycles: Crank angle distribution, thigh angles evolution, power developed on the rear wheel and stimulation onset for both legs (Figure 5). The estimated average cycling velocity for the trial presented in this figure was of 5 km/h based on home trainer data, with a current intensity manually set by the participant of 112.5 mA. By observing power distributions over the crank cycles, we could validate the stimulation onset was adequate regarding the power produced. The positive power recorded by the torquemeter corresponded to the actual power produced by the participant, while the negative power corresponded to inertia. The average power was low because, in this session, the pilot was mainly entertaining the cycling movement by taking advantage of inertia and producing power to counteract the losses due to friction.

**Figure 6:**
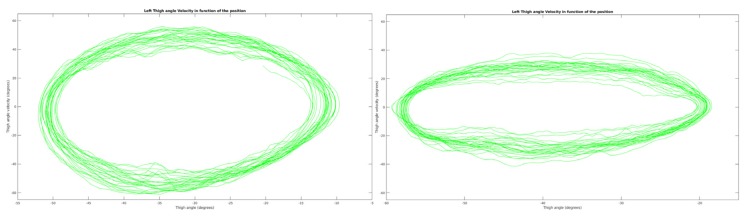
Representation of the left thigh angular velocity as a function of angular position. (**Left**) 27 cycles in stationary condition. (**Right**) 25 cycles in over-ground condition.

The cyclic behavior can be appreciated by plotting the angular velocity as a function of the angular position (Figure 6).

The exact same set of parameters was used for over-ground and stationary cycling. In stationary conditions the thigh angles range was [−50°, −10°] and angular velocity range [−60°, 56°]. In over-ground conditions the thigh angles range was [−60°, −20°] and angular velocity range [−40°, 40°]. The thigh angle was relative to the horizontal, which explains the shift in values due to different trike inclinations on the home-trainer and flat surface, despite the same 40° range of motion. The cycling velocity was lower in over-ground conditions compared to stationary, as the resistive forces were higher. Furthermore, the trial was performed at the end of the session and some muscular fatigue was already influencing the performance.

## 4. Discussion and Conclusions

This study aimed at investigating and developing a new method to simplify the design of stimulation patterns in FES-induced cycling, without requiring any sensor installation on the recumbent trike used. The goal was to demonstrate the feasibility of further simplifying existing approaches for setting any tricycle as an FES-cycling device for an individual with complete SCI, without any prior stimulation timing pattern tuning, nor adaptation due to the bike geometry or the size and position of the pilot, using only two inertial sensors. The present case-study experiment intends to be a proof of concept of this new control modality.

In our experience of FES-cycling, adjusting the stimulation timing of the different muscles to be activated using crank angle is time consuming and the optimization regarding power consumption may be complex. The pattern needs to be adapted for different situations: Sitting position, leg length, bike geometry, etc. We believe it is more intuitive and generalizable to define triggering events relative to the pilot instead of the bike. Setting a stimulation onset at a predefined crank angle cannot be translated to multiple bikes or pilots. When tuning a crank-angle-based stimulation pattern, it is necessary to manually correlate each crank value to the pilot oneself; the users take into account the seat height compared to the crank, the lower limb lengths and possible asymmetry, the seat tilting, the length of the boom, and the length of the crank handles. If the position of the pilot varies, the stimulation pattern needs to be entirely defined again.

Detecting peak knee angles was initially intended as a stimulation event. Meanwhile, depending on each individual, when the peak knee flexion is reached over the crank cycle, the hip flexion could modify the starting event regarding the seat height compared to the crank, it also requires more sensors. We have observed that the time when the pedal crank reached few degrees before its vertical position corresponds to the time when the thigh reaches its maximum tilting angle. This event detection was supposed to be the same no matter the leg length or bike geometry while enabling to start the stimulation a few degrees before the pedal was vertical, in order to obtain the best muscle mechanical response at the optimal timing. Therefore, the hypothesis was made of detecting this event as the simplest and universal stimulation onset event. Furthermore, this can be done using only two inertial sensors.

The result obtained here in one participant confirmed the feasibility of this very simple approach compared to the state-of-the-art, i.e., crank-angle-based stimulation patterns. Further work is needed to validate the approach with other participants.

More refined control laws may be considered to optimize cycling performances and robustness [25], but the proposed controller has the great advantage of being simple with no a priori calibration phase.

In this study, only the quadriceps were stimulated. A more efficient pedaling could be achieved by adding hamstrings and glutei stimulation, but could require the need to define other detectable stimulation events.

Using the instrumented home trainer, we observed that the effective mechanical response of the quadriceps stimulation associated to the pushing phase was less than a 200-ms duration at 47 rpm (cadency used for our participation to the Cybathlon competition [32]). This observation highlights the need for a more accurate method able to monitor the actual power produced and adapt the corresponding stimulation timing event relatively to the PTF. This would need to accurately measure the weak power developed using dedicated force pedals during over-ground cycling.

Automatically adjusting the stimulation parameters to counteract muscle fatigue or ground surface changes remains an open problem.

A video of the experiment is available online here: https://youtu.be/gbgGxbBD-VM.
